# Medical Items

**Published:** 1897-06

**Authors:** 


### *MEDICAL ITEMS.*

---

Dr. J. C. Davenport, of Fairburn, Ga., died May 16, aged 70 years.

---

Dr. A. E. Oglesby, of Reidsville, Ga., was fatally shot by a step-son, May 24th.

---

Senn says the essential features of the modern treatment of malignant tumors may be summed up as follows: Operate early and thoroughly. The treatment of unoperable sarcoma by injections of the sterilized toxins of the streptococcus of erysipelas and the bacillus prodigiosus has not had the expected results."—*Ex.*

---

The *American Journal of Dermatology and Genito-Urinary Diseases* is the name of a new journal published in St. Louis, in the interest of those specialties. Editor and owner, Dr. S. C. Martin. The journal makes a very creditable beginning in its initial number (April), and we trust that it will succeed.

---

Thirteen well-known physicians in the South are to be tried at Atlanta, Ga., by a Medical Board on a charge of breach of ethics in permitting their pictures to be printed in the newspapers.—*Western Medical Review* (St. Louis).

The members of the medical profession of Atlanta, Ga., are much exercised over the impending trial before a medical court of thirteen of the best-known physicians in the South on a charge of permitting their pictures to be printed in the newspapers.—*Am. Medico-Surg. Bulletin.*

What great "medical court" is this in Atlanta, with authority over physicians of the South? We have never heard of any such. There has been no such trial here and none is impending. Our misguided contemporaries are either badly mixed or they are victims of the fake-dispatch fiend.

WE acknowledge receipt of booklet entitled "The Mineral Waters of Mt. Clemens, Michigan," by Dr. Richard Leuschner, Resident Physician. It endeavors to furnish correct information as to the nature and constituents of the famous springs at Mt. Clemens, to explain their physical action, to designate the several kinds of diseases for which these baths should be given, and finally to give suitable directions as to how the waters are most beneficially employed. The author will send copies to physicians upon application.

---

THE annual commencement of the Medical College of Alabama took place at Mobile on the evening of April 9, and was of particular interest from the fact that the union between this institution and the University of Alabama was then formally ratified. The University has had a long and honorable career, having been founded in 1827, and the college, which was organized in 1860, numbers many of the most eminent practitioners of the South among its alumni. This union of forces will redound to the advantage and usefulness of both, and is another indication of the general trend of effort in different portions of the country to concentrate means and abilities towards the attainment of higher education.—*Medical News.*

---

WE acknowledge with thanks invitation to attend the sixty-fifth annual meeting of the British Medical Association, which will be held at Montreal, August 21st, September 1st, 2d and 3d. A large and enthusiastic meeting of the Association is expected. Addresses will be delivered as follows: *Medicine,* Dr. William Osler, of Baltimore; *Surgery,* Mr. T. Mitchell Banks, of Liverpool; *Public Medicine,* Dr. Herman M. Biggs, of New York. The scientific business of the Association will be conducted in eleven sections—Medicine, Surgery, Public Medicine, Obstetrics and Gynecology, Pharmacology and Therapeutics, Pathology and Bacteriology, Psychology, Ophthalmology, Laryngology and Otology, Anatomy and Physiology, and Dermatology. A most inviting program has been prepared, which includes a number of amusement features, garden parties, excursions, conversaziones, etc., etc.

